# Characterization of *Streptomyces* Isolates Associated with Estuarine Fish *Chanos chanos* and Profiling of Their Antibacterial Metabolites-Crude-Extract

**DOI:** 10.1155/2020/8851947

**Published:** 2020-09-23

**Authors:** Muhammad A. Kurnianto, Harsi D. Kusumaningrum, Hanifah N. Lioe

**Affiliations:** ^1^Food Science Study Program, Graduate School, IPB University, Bogor, Indonesia; ^2^Department of Food Science and Technology, Faculty of Agricultural Engineering and Technology, IPB University, Bogor, Indonesia

## Abstract

*Streptomyces* has been reported as an essential producer of bioactive substances, including antibiotics and other types of antimicrobials. This study investigated antibacterial-producing *Streptomyces* isolated from the gut of estuarine fish *Chanos chanos*, emphasizing screening for the producer of peptide-containing antibacterial compounds. Eighteen isolates were found during preliminary screening, in which four isolates showed the best antibacterial activities. Based on the morphological, physiological, and biochemical characterization, as well as 16S rRNA partial sequencing, all of the four isolates belonged to *Streptomyces*. Three isolates were suspected as novel isolate candidates based on homology presentations and phylogenetic tree analysis. Disk-diffusion assay of the metabolite-crude-extract from the isolates showed broad-spectrum inhibitory activities against *Staphylococcus aureus* ATCC 25923, *Bacillus cereus* ATCC 10876, *Escherichia coli* ATCC 25922, and *Pseudomonas aeruginosa* InaCC B52 with minimum inhibitory concentration and minimum bactericidal concentration ranging from 2.5–10 mg/mL and 5–10 mg/mL, respectively. The highest antibacterial activity with low MIC and MBC values was shown by isolate AIA-10. Qualitative HPLC profiling revealed that the metabolic-crude-extracts showed many peaks with intensive area at 210 and 214 nm, especially from SCA-11 and AIA-10, indicating the presence of peptide groups in the structure of the constituent compound. The results also suggested that crude extracts SCA-11 and AIA-10 had higher hydrophobicity properties than the other extracts. Further characterization of the active compound was needed to find out which compounds were responsible for the antibacterial activity. The results of this study indicated that some *Streptomyces* isolated from new environmental niches, i.e., gut of estuarine fish *Chanos chanos*, produce promising peptide-containing bioactive compounds.

## 1. Introduction

*Streptomyces* is one of the organisms with a complex life cycle that has been widely explored in its ability to produce bioactive compounds [1]. In general, *Streptomyces* are considered as free-living bacteria and are commonly found in terrestrial ecosystems. However, researchers' increased focus on these bacteria revealed that they could associate or form a symbiosis with eukaryotic hosts and have the most extensive relative abundance throughout the ecosystem [2–5]. This reflects their physiological ability and gives reciprocal effects of evolution that affect the growth, reproduction, and capacity of bacteria to produce a variety of secondary metabolites [6–8]. This condition provides an excellent reason to promote research in this emerging area [3].

Estuarine is one of the aquatic ecosystems of *Streptomyces* habitat that is rarely explored. In this ecosystem, there is conflux between the saltwater from sea and freshwater from rivers that causes continual change in salinity levels and persistent tidal gradients [9–11]. This condition also makes the estuary rich in organic material that can support the growth of various communities of the microorganism [12]. But, on the other hand, it also triggers competition between organisms over nutrition and encourages some organisms, such as bacteria, to produce secondary metabolites as a defence response [13]. Adaptation in this competition encourages the formation of special physiological and metabolic abilities that enable them to produce different types of secondary metabolites that cannot be produced by bacteria living in normal conditions [14].

The most intriguing and widely explored characteristic of *Streptomyces* is its ability to produce bioactive secondary metabolites with antimicrobial activity, one of which is peptide-containing antibacterial [1, 15, 16]. Peptides become one of the structural classes of antimicrobials produced from ribosomes or nonribosomes [16]. The ribosomally synthesized and post-translationally modified peptides (RiPPs) are a large class of natural products introduced by post-translational modifications with a high degree of structural diversity and a wide variety of bioactivities that are poorly understood [16, 17]. Some RiPPs which are produced by *Streptomyces* have been reported, such as siamycin-I by *Streptomyces* sp. [18], cinnamycin by *S. cinnamoneus* [19], bottromycin by *S. bottropensis* [20], bacteriocin-like by *S. scopuliridis* [21], grisemycin by *S. griseus* [22], telomestatin by *S. anulatus* [23], and archromosin by *S. achromogenes* [24].

A preliminary study by Kurnianto et al. [25] reported isolation of 22 *Actinobacteria* isolates from estuarine milkfish (*Chanos chanos*) and blue-spot mullet fish (*Mugil cephalus*) based on their inhibitory index against four test bacteria. This study investigated selected *Streptomyces* isolates associated with milkfish *Chanos chanos* that possibly produce peptide-containing metabolite with potential antibacterial activity. This metabolite was explored to look for possible uses in food in the future. HPLC profiling of the metabolite-crude-extract was carried out to figure out the spectra of the target group.

## 2. Materials and Methods

### 2.1. Preliminary Screening of Potent Isolates as Antibacterial Producers

Twenty-eight presumptive *Streptomyces* isolates from the milkfish gut from the previous study [25] were purified, regrown on yeast-malt extract agar (ISP-2 agar; yeast extract 4 g, malt extract 10 g, dextrose broth 4 g, bacteriological agar 20 g, and distilled water 1000 mL), and incubated for 10–14 days at 30°C. The isolates were, then, screened qualitatively based on their inhibition zone using cross streak and, later, quantitatively by the double-layer diffusion method against pathogenic bacteria. Four different bacteria were used as indicators, i.e., Gram-positive (*Bacillus cereus* ATCC 10876 and *Staphylococcus aureus* ATCC 25922) and Gram-negative (*Escherichia coli* ATCC 25922 and *Pseudomonas aeruginosa* InaCC B52). In the cross-streak method, a Mueller-Hinton agar (Oxoid, UK) plate was scratched with the isolate in the center of the plate and incubated for 7 days at 30°C. The indicators were, then, scratched perpendicular to the first scratch and incubated at 37°C overnight [26]. In the double-layer diffusion method, the isolates that had been grown for seven days on ISP-2 agar were cut using a cork borer (6 mm diameter) and placed in a plate containing two-layers of nutrient agar (NA; Oxoid, UK), which had been inoculated with indicators on the top layer. The bottom layer was NA with a single-strength concentration (28 g NA in 1000 mL destination water), and the top layer was NA with a half-strength concentration (14 g NA in 100 mL distillation water). Plates were incubated at 37°C overnight [27]. Isolates with the best inhibitory activity against indicators were selected for further analysis.

### 2.2. Molecular Characterization Using 16S rRNA Partial Sequencing

The presumptive *Streptomyces* isolates were grown in yeast-malt extract broth (ISP-2 broth; yeast extract: 4 g, malt extract: 10 g, dextrose: 4 g, and distilled water 1000 mL) for 7 days at 30°C. The culture media were, then, centrifuged (Hermle, Germany) at 10,000 rpm for 15 minutes to obtain cell biomass (mycelia) used for DNA extraction processes [28]. Furthermore, the 16S ribosomal RNA gene was amplified by PCR using 9F (5′-GAGTTTGATCCTGGCTCAG) and 1541R (5′-AAGGAGGTGATCCAGCC) primers [29]. The product amplified by PCR was qualitatively evaluated by electrophoresis 1% (w/v), and then, it was sequenced. The sequenced data were converted to the FASTA format and analyzed using the BLAST program to identify homology and obtain similar isolates that are closely related based on the database of NCBI (http://www.ncbi.nlm.nih.gov). FASTA files from presumptive *Streptomyces* and similar closely related isolates were aligned using ClustalX, and their phylogenetic trees were analyzed using MEGA 6 with Neighbor-Joining (NJ) algorithm with 1000 bootstrap resampling [30].

### 2.3. Morphological, Cultural, Physiological, and Biochemical Characterization

Macroscopic morphology was studied by inoculating isolates on four different media, i.e., starch casein agar (SCA; Starch 10 g, K_2_HPO_4_ 2 g, KNO_3_ 2 g, casein 0.3 g, MgSO_4_.7H_2_O 0.05 g, CaCO_3_ 0.02 g, FeSO_4_.7H_2_O 0.01 g, bacteriological agar 15 g, and distilled water 1000 mL), yeast-malt agar (ISP-2 agar), tryptone-yeast agar extract (ISP-1; tryptone 5 g, yeast extract 3 g, and distilled water 1000 mL), and inorganic salts-starch agar (ISP-4; K_2_HPO_4_ 1 g, MgSO_4_.7H_2_O 1 g, NaCl 1 g, (NH_4_)_2_SO_4_ 2 g, CaCO_3_ 2 g, trace salts solution 1.0 mL, and distilled water 500 mL). After incubation, three characteristics were observed, i.e., pigment production, aerial mycelium, and substrate mycelium [26]. Microscopic morphology was studied using the coverslip method by which spore-bearing hyphae morphology with the entire spore chain was observed under a light microscope (Nikon, Japan) as described by Shirling and Gottlieb [31]. The utilization of carbohydrate sources was determined by growing each isolate on media that were supplemented with carbon sources at a concentration of 1% [32]. The ability of isolates to tolerate pH and NaCl was determined by inoculating isolates in an inorganic salt-starch agar medium (ISP-4) conditioned at a pH range of 5–10 and NaCl concentrations of 2–6% [33]. The ability of isolates to produce hemolysin, which can break down red blood cells, was determined by inoculating the isolates on sheep blood agar [34]. The ability to produce specific enzymes and other biochemical tests was evaluated using the API 20 kit (Biomerieux, France) [35, 36]. All results were collected after 7 days of incubation at 30°C.

### 2.4. Determination of the Optimum Production Medium and Production of the Metabolite-Crude-Extract

The determination of the optimum production medium was conducted by culturing the isolates on three different media: yeast-malt extract broth (ISP-2 broth), nutrient broth glucose (NBG; nutrient broth 13 g, glucose 4 g, and distilled water 1000 mL), and Gause synthetic broth (GSB; starch 20 g, NaCl 0.5 g, FeSO_4_.H_2_O 0.01 g, KNO_3_ 1 g, K_2_HPO_4_ 0.5 g, MgSO_4_ 0.5 g, and distilled water 1000 mL). The culture media were incubated for 10 days at 30°C and, then, centrifuged (Hermle, Germany) at 8000 rpm for 15 minutes to separate the supernatant and biomass. The supernatant was evaluated for antibacterial activity by the microdilution method [37], against *Staphylococcus aureus* ATCC 25922 and *Escherichia coli* ATCC 25922. Optimum production media were determined by the better ability of the culture supernatant to inhibit the growth of test bacteria. The lower optical density (OD) found in the microdilution test indicated the higher inhibitory activity. The dry weight of the cell biomass and antibacterial activity, starting on the 3rd day until the 11th day (every 48-hours interval), were also measured to determine the growth phase of bacteria and the optimum antibacterial production time [14, 37].

The metabolite production was carried out by inoculating the isolates on the best growth media for optimum incubation period in a shaker incubator (New Brunchwick, Germany) at 120 rpm at 30°C. The bacterial culture was centrifuged (Hermle, Germany) at 8000 rpm for 15 minutes. Cell-free supernatants obtained were, then, mixed with ethyl acetate (Merck, Germany) 1 : 1 (v/v) followed by the extraction with a separating funnel and evaporated using a rotary vacuum evaporator (Buchi, Switzerland) at 45°C to obtain the metabolite-crude-extract. The dried metabolite-crude-extract was dissolved in DMSO (Merck, Germany), and a stock concentration was prepared of about 10 mg/mL [26, 38].

### 2.5. Antibacterial Activity Test and Determination of the Minimum Inhibitory Concentration (MIC) and Minimum Bactericidal Concentration (MBC)

A sterile disc (6 mm diameter; Oxoid, UK) was dripped with 20 *μ*L of the metabolite-crude-extract, placed on the surface of the Mueller-Hinton Agar (MHA; Oxoid, UK) that has been rubbed with a cotton swab containing test bacteria, and then, incubated for 24 hours at 37°C. The antibacterial activity was, then, assessed by measuring the inhibition zone diameter (mm) on agar [39]. The determination of MIC values of the metabolite-crude-extract was performed by the microdilution method. The metabolite-crude-extracts with a concentration of 10, 5.0, 2.5, 1.25, 0.625, 0.312, and 0.156 mg/mL were added to microtiter plate which contained 0.1 mL Mueller-Hinton Broth (MHB; Himedia, India). An amount of 0.1 mL of the test bacteria was pipetted into each well, to obtain a final inoculum density obtained of approximately 1 × 10^6^ CFU/mL. Afterward, the microtiter plate was incubated for 24 hours at 37°C. The minimum bactericidal concentration (MBC) was determined by taking a small amount of mixed liquid in a microtiter plate from the MIC test results with a loop, then scratched on Mueller-Hinton Agar (MHA; Himedia, India), and incubated for 24 hours at 37°C. All analyses used ampicillin (Oxoid, UK) as a positive control and DMSO (Merck, Germany) as the negative control [40].

### 2.6. Profiling of the Metabolite-Crude-Extract Using HPLC

The metabolite-crude-extract was dissolved in methanol (1 mg/mL; Merck, Germany) and, then, analyzed by using the Agilent 1200 Series HPLC using the 4.60 × 150 mm (5 *µ*m) Zorbax eclipse XDC-C18 column. The HPLC system used an elution with a gradient mobile phase (A acetonitrile (Merck, Germany), B water; 0–30 min 10% A–50% B). The sample was run in 30 minutes at a flow rate of 0.5 mL/min. The compounds present were detected using a multiwavelength UV-Vis detector (MWD) at 210, 214, 254, and 276 nm [41].

## 3. Results

### 3.1. Potential Isolates as Antibacterial Producers

**Figure 1:**
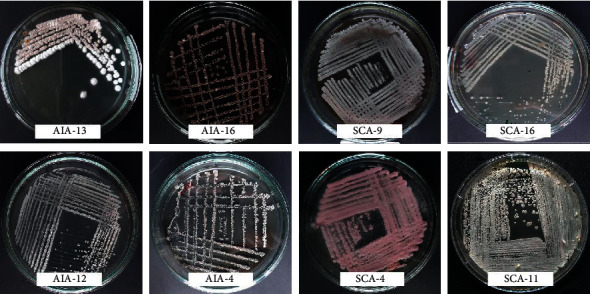
Morphological diversity of presumptive *Streptomyces* isolates in yeast-malt extract agar (ISP-2 agar).

The morphological appearance of some purified isolates on ISP-2 agar is shown in Figure 1. Most of the isolates showed characteristics such as a white-colored colony surface and a dry and powdered texture, and some colonies were able to produce pigments.

Preliminary screening using the cross-streak method indicated that 23 of 28 isolates showed inhibitory activity against, at least, one indicator. Meanwhile, the evaluation using a double-layer diffusion agar method showed only 18 isolates which had inhibitory activity (Table 1). Out of them, four isolates (SCA-5, SCA-8, AIA-10, and SCA-11) exhibited potential inhibitory activity against all indicators.

### 3.2. Characteristics of Selected *Streptomyces* Isolates

**Figure 2:**
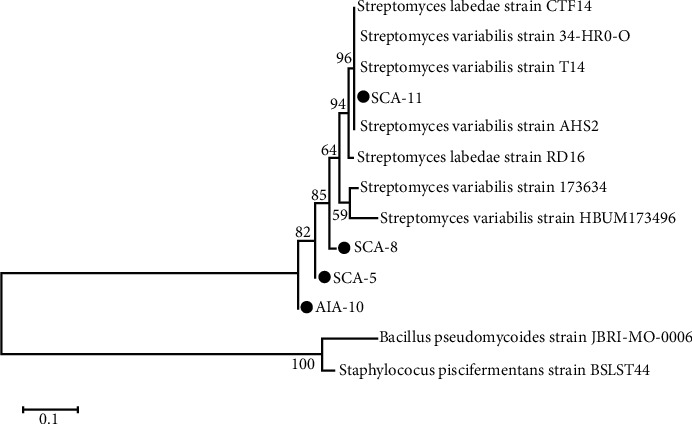
Phylogenetic tree of SCA-5, SCA-8, SCA-11, and AIA-10 constructed with the aid of MEGA 6.0 program.

The molecular characterization based on 16S rRNA partial sequencing indicated that all isolates were *Streptomyces* (Table 2). Isolate SCA-11 showed maximum 16S rRNA gene sequence homology (100%) with *Streptomyces variabilis* strain 34-HR0-O, T4, and AHS2. However, other three isolates (SCA-5, SCA-8, and AIA-10) showed 16S rRNA gene sequence homology below 98%, in which SCA-5 and AIA-10 were homologous to *Streptomyces variabilis* strain HBUM173496 and SCA-8 was homologous to *Streptomyces labedae* strain RD16. The phylogenetic analysis results also showed that the three isolates were separated from their closest strains. The phylogenetic tree construction is shown in Figure 2.

Morphological characterization showed that all isolates were Gram-positive filamentous bacteria. Morphological characterization on four different media showed an efficient growth on ISP-2 media (Table 3). On these media, the four selected isolates formed aerial mycelium with white to grey colour and substrate mycelium with white to brown colour. One of the isolates, SCA-8, showed the ability to produce light-yellow pigments that were resulted from the colour changes in the growth media.

**Figure 3:**
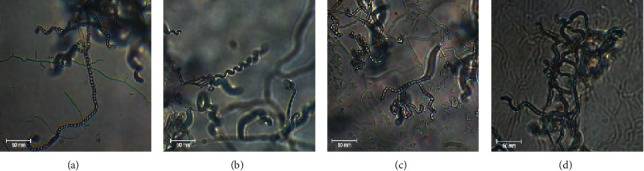
Microscopic morphology of selected *Streptomyces* examined by using a light microscope (1000×): (a) SCA-5, (b) SCA-8, (c) SCA-11, and (d) AIA-10.

The sporophore morphology observation by a light microscopic analysis showed that the isolates had retina-culiaperti, rectus-flexibilis, and spira spore chain types (Figure 3).

The microscopy morphological, physiological, and biochemical characteristics of the selected isolates are presented in Table 4. Tolerance tests on pH and NaCl showed that all isolates were able to grow well at pH 6 to 9, with NaCl concentrations reaching 6%. The majority of isolates were also able to utilize carbon sources such as sucrose, glucose, amygdalin, and arabinose and were able to produce urease and hydrogen sulphide.

### 3.3. Optimum Production Medium

**Figure 4:**
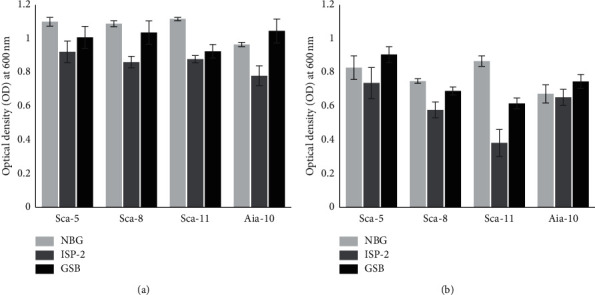
The OD values at 600 nm of (a) *Escherichia coli* and (b) *Staphylococcus aureus* growth exposed to the supernatant of isolates that were grown in NBG (nutrient broth glucose) broth, ISP-2 (yeast-malt extract) broth, and GSB (Gause synthetic) broth.

The determination of antibacterial production media was carried out to determine the best medium for growth and to maximize the production of antibacterial compounds. The results showed ISP-2 as the best production medium. The inhibitory activity test using the microdilution method showed that exposure to the ISP-2 supernatant resulted in lower optical density (OD) than exposure to other media supernatants. Statistical analysis also showed a significant difference in OD *E. coli* exposed to the ISP-2 supernatant compared to OD *E. coli* exposed to other media supernatants (all supernatant isolates). Different results were obtained for *S. aureus*, which was only in the supernatant exposure of ISP-2 from SCA-11 and SCA-8 isolates which had significantly different OD values (Figure 4).

**Figure 5:**
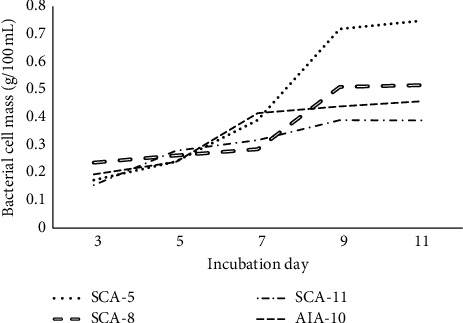
Growth curve of selected *Streptomyces* isolates on ISP-2 broth based on incubation times; mycelial pellets were harvested for growth determination by biomass measurement.

The growth curves based on the dry weight of cell biomass of all isolates that were produced in ISP-2 broth (selected optimum media) are shown in Figure 5. Three isolates (SCA-5, SCA-11, and AIA-10) started their logarithmic phase within the 5th day after inoculation. This phase generally continued until the 9th day, and after that, the stationary phase occurred. Different from the other three isolates, SCA-8 started the logarithmic phase at the 7th day, continued until the 9th day, and then, proceeded to the stationary phase. Based on the microdilution test of the cell-free supernatant, all isolates likely started to produce metabolites that were able to inhibit the growth of test bacteria after the 5th day of incubation. The inhibitory activity continued to increase until the 9th day or after proceeding to the stationary phase (data not shown).

### 3.4. Antibacterial Activity of the Metabolite-Crude-Extract

All metabolite-crude-extracts produced by selected isolates showed the promising result of antibacterial activity. The highest antibacterial activity was demonstrated by the AIA-10 crude extract with the highest inhibitory zone of 22.2 mm against *S. aureus* and 21.3 mm against *P. aeruginosa*. The antibacterial activity of AIA-10 was significantly higher compared to the other metabolite-crude-extracts (Table 5).

The MIC values of the metabolite-crude-extract of all test bacteria ranged from 2.5 to 10 mg/mL. Meanwhile, the MBC values ranged from 5 to 10 mg/mL (Table 6). The highest MIC and MBC were exhibited by AIA-10 and SCA-11 with values of 2.5 mg/mL and 5 mg/mL, respectively. These results showed that the MBC value of all metabolite-crude-extracts was two times higher than the MIC value, except for SCA-8.

### 3.5. HPLC Metabolite-Crude-Extract Profile

**Figure 6:**
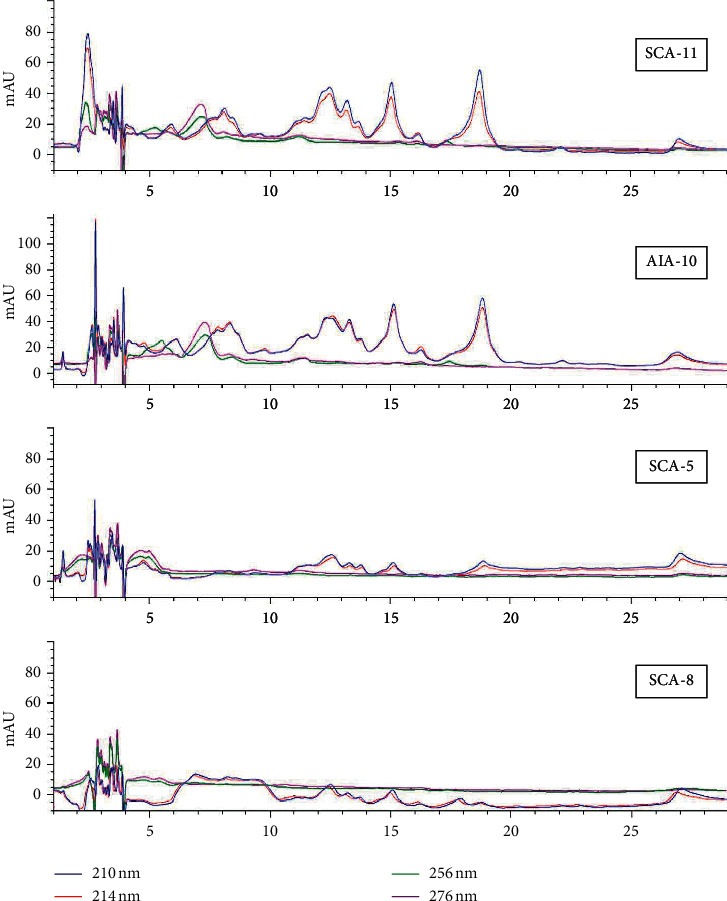
HPLC metabolite-crude-extract profile from four *Streptomyces* isolates detected at 210, 214, 254, and 276 nm.

The chromatogram profile of all metabolite-crude extracts exhibited many peaks detected during a retention time of 0–30 minutes. Qualitatively, the metabolite-crude-extracts showed similar trends at 210 with 214 nm and 254 with 276 nm. At 210 and 214 nm, 10 peaks, 11 peaks, 9 peaks, and 9 peaks were detected on the chromatogram of SCA-11, AIA-10, SCA-5, and SCA-8, respectively. Meanwhile at 254 nm, 6 peaks, 8 peaks, 3 peaks, and 4 peaks were detected in the chromatogram of SCA-11, AIA-10, SCA-5, and SCA-8, respectively. At 276 nm, less peaks were relatively detected, i.e., 4 peaks at SCA-11, 6 peaks at AIA-10, 3 peaks at SCA-5, and 3 peaks at SCA-8. Based on this trend, it was known that at 210 and 214 nm were optimal absorption occurred, in which all metabolite-crude-extracts exhibited more peak numbers with more intensive areas, especially isolate SCA-11 and AIA-10. Furthermore, peaks with intensive areas were mostly detected at the beginning and the middle of the chromatogram or in the retention time between 0–5 min, 6–9 min, and 11–20 min. Meanwhile, at 254 and 276 nm, the majority of peaks were detected in the initial 5 min retention time with smaller areas. Specifically, metabolites-crude-extract SCA-11 and AIA-10 exhibited identical peaks with different intensities at a retention time of 7.189 minutes (Figure 6).

## 4. Discussion

Four of 18 isolates with the highest antibacterial activity had been selected and characterized. Three isolates were confirmed as *S. variabilis* and one isolate as *S. labedae*. Dholakiya et al. [14] reported that *S. variabilis* RD-5 formed white and brown-white aerial and substrate mycelium characteristics and produced yellowish pigments. Meanwhile, Mangamuri et al. [42] reported that *S. labedae* VSM-6 formed greyish-white and brown aerial and mycelium substrate characteristics and produced a spiral type spore chain. Furthermore, phylogenetic tree construction and alignment carried out of all nucleotide sequences in this study show that three of four isolates occupied phylogenetic positions separated from their closest group and showed homology values of less than 98%. These results indicated the possibility of the three isolates to belong in a different species or considered as new species. Zhi et al. [43] stated that the percentage of homology with closest relatives was less than 98%, indicating the possibility of different species. Hagstrom et al. [44] also stated that the isolate with the sequence homology percentage ≥97% can represent identical species, the sequence homology percentage between 93 and 97% indicated the same identity at the genus level, but differ at the species level, and the homology percentage <93% indicated novelty at the genus level. However, claims regarding this matter must be further confirmed to validate the precise taxonomic position.

Carbon and nitrogen sources in the medium likely affected the antibacterial production [45]. The determination of media for antibacterial production showed that ISP-2 broth was the best medium. ISP-2 broth contained glucose as a simple carbon source and malt and yeast extract as organic nitrogen sources which can increase the ability of growth, pigment production, and the production of antibacterial agents [46, 47]. Al Farraj et al. [48] reported that the level of 0.55% glucose and 0.835% yeast extract in the production medium was the optimum level of bioactive metabolites production with maximum antibacterial activity *Streptomyces* sp. AS4. Another study by Abdelghani [49] also reported that malt extract concentration of 1.0% (w/v) showed maximum antibacterial yield in *S. albovinaceus*. A study of cell biomass and antibacterial activity of all isolates showed a positive correlation, which was the optimum antibacterial activity found in the incubation period of days 9 to 11 or when the growth reached the stationary phase (maximum cell biomass). Previous studies reported that the antibacterial activity of *S. variabilis* RD-5 increased with increasing cell growth. *S. variabilis* RD-5 showed optimum antibacterial activity on days 7 and 8 or at the end of the second (stationary) growth phase [14]. According to Singh et al. [33], maximum antimicrobial activity was achieved after reaching the maximum value of biomass.

The metabolite-crude-extract from all isolates was tested and showed broad-spectrum antibacterial activity with the highest inhibitory zone and MIC and MBC values shown by AIA-10 crude extracts on *S. aureus* and *P. aeruginosa*. *Actinobacteria* isolates from fish gut digesta showed a result similar to that in the previous study, i.e., their crude extracts demonstrated broad-spectrum antibacterial activity against *E. coli, S. aureus, B. subtilis, B. cereus, Streptococcus agalactiae*, and *V. mimicus* [25, 50, 51]. Further identification of the metabolite-crude-extract found sebastenoic acid as the active component responsible for antibacterial activity with MIC values ranging from 10.0 to 110.6 *µ*g/mL [51]. The difference in antibacterial activity between the test bacteria was likely due to differences in the structure and composition of the test bacteria's cell wall. In Gram-positive, peptidoglycan polymers are very close to the cell surface, allowing antibacterial compounds to penetrate cells easily. This is different from Gram-negative because it has an outer membrane consisting of lipopolysaccharides, which act as a barrier to hydrophobic and hydrophilic compounds with certain molecular weights [52]. According to Choi and Lee [53], several types of antibacterial, which are hydrophilic with a small molecular weight (<600 Da), can penetrate the outer membrane by diffusing through aqueous channels formed by proteins called porins. Based on this review, it was suspected that the active component with antibacterial properties contained in crude extracts had a molecular weight of less than 600 Da.

The study reported that the profiles of all metabolite extracts at the absorption band 210 nm were best describing the metabolites. The absorption band's range suggested the presence of amido-chromophoric groups containing one or more peptide bonds in their molecules [54, 55]. Strong absorption of this absorption range was likely due to the presence of the amido-chromophoric groups in the compound led to the *π* ⟶ *π*^∗^ or *n* ⟶ *π*^∗^ excitation of electrons [55]. Specifically, in AIA-10 and SCA-11, it was also thought to have an aromatic moiety on its molecule structure because a typical peak was detected at 254 and 276 nm [54, 56]. A similar study reported by Ezra et al. [54] obtained peaks at 208 and 214 nm and a broad band at 270 on the coronamycin antimicrobial peptide. Further analysis identified that the peaks found between 208 and 214 nm were composed of threonine, alfa-aminobutyric acid, methionine, and leucine residues, while the 270 nm broad band was corresponded to tyrosine residue [54]. The crude extract profile also showed that SCA-11 and AIA-10 likely exhibited higher hydrophobicity properties than the other two extracts, as shown by the presence of peak with an intensive area at the retention period of 11–20 min. These properties reported could increase antibacterial activity through hydrophobic interaction of antibacterial compounds in the form of insertion into cell membranes and, then, disrupt intracellular bacteria [57, 58]. However, other factors such as charge, size, sequence, and structure of the compound also influenced the antimicrobial properties [59]. Purifying of the metabolite-crude-extract, therefore, had been subjected for further study to identify and confirm the factors responsible for these biological activities.

## 5. Conclusions

The four isolates from the gut of estuarine fish *Chanos chanos* which were identified as *Streptomyces* showed potential inhibitory activity against test bacteria on preliminary screening. Production with optimum medium ISP-2 broth, extraction, and antibacterial activity test on the metabolite-crude-extract showed that all isolates had broad-spectrum antibacterial activity against *S. aureus, B. cereus, P. aeruginosa*, and *E. coli.* Compounds suspected to be peptide in a metabolite-crude-extract are thought to be responsible for antibacterial activity. These results indicate that *Streptomyces* isolated from new environmental niches produce potential bioactive compounds and facilitate the discovery of new antibacterial agents.

## Figures and Tables

**Table 1:** Preliminary screening using cross streak and evaluation of antibacterial activity using double-layer agar diffusion.

Isolates	Inhibition zone (mm)							
	*S. aureus*		*B. cereus*		*E. coli*		*P. aeruginosa*	
	Cross streak	Double-layer agar	Cross streak	Double-layer agar	Cross streak	Double-layer agar	Cross streak	Double-layer agar
SCA-7	−	−	+++	12.9 ± 0.47	−	−	−	−
SCA-6	++	11.6 ± 0.72	−	−	−	−	−	−
SCA-13	+++	14.6 ± 0.70	++	−	−	−	−	−
AIA-1	−	−	++	−	−	−	−	−
AIA-6	−	−	++	−	−	−	−	−
AIA-5	+	−	−	−	−	−	−	−
SCA-4	−	−	++	−	+	−	−	−
SCA-10	−	17.9 ± 0.50	−	−	−	−	−	−
SCA-9	−	20.6 ± 0.37	−	−	++	−	+	−
SCA-5	+++	19.5 ± 0.32	+++	10. 9 ± 0.28	++	13.5 ± 0.83	+	−
SCA-11	+++	20.6 ± 0.53	+++	9.8 ± 0.09	+++	14.9 ± 0.63	++	10.3 ± 0.64
AIA-12	+++	16.3 ± 0.77	+++	−	++	−	−	−
AIA-13	−	13.8 ± 0.49	+++	9.5 ± 0.06	−	−	−	−
SCA-12	+	10.6 ± 0.49	+	−	++	−	−	−
AIA-10	+++	21.4 ± 1.47	++	14.3 ± 0.62	+++	13.3 ± 1.04	+	−
AIA-9	−	13.0 ± 0.11	−	−	−	−	−	−
AIA-15	−	−	+	−	−	−	−	−
AIA-16	−	14.2 ± 0.09	++	−	+	−	−	−
SCA-8	+++	18.8 ± 0.14	++	12.2 ± 0.40	++	16.3 ± 1.24	+	−
AIA-17	++	18.7 ± 0.24	++	−	++	8.6 ± 0.47	−	−
SCA-16	−	−	−	−	−	−	+	−
SCA-17	−	−	−	−	−	−	++	−
SCA-18	−	9.9 ± 0.57	−	−	−	−	−	−
SCA-19	−	13.5 ± 1.61	−	−	−	−	−	−
SCA-20	+	−	−	−	−	−	−	−
SCA-21	−	−	+	−	−	−	−	−
SCA-22	−	15.9 ± 0.29	−	9.4 ± 0.26	−	−	−	−
SCA-23	−	−	+	−	−	−	−	−

(−): no inhibition; (+): weak inhibition; (++): moderate inhibition; (+++): good inhibition; value was mean ± SD (standard deviation) from three replication; (−): no zone of inhibition.

**Table 2:** The results of gene sequence alignments of 16S rRNA selected *Streptomyces* isolates to data available at NCBI (BLASTN).

Isolates	BLAST result (16S ribosomal RNA gene, partial sequence)	Max score	Total score	Query cover (%)	E value	Ident (%)	Accession
SCA-5	*Streptomyces variabilis* strain HBUM173496	942	942	99	0	93.92	EU841661.1
SCA-8	*Streptomyces labedae* strain RD16	652	652	99	0	95.37	KY378908.1
SCA-11	*Streptomyces variabilis* strain 34-HR0-O	1454	1454	100	0	100	MF077022.1
	*Streptomyces variabilis* strain T14	1454	1454	100	0	100	KY213667.1
	*Streptomyces variabilis* strain AHS2	1454	1454	100	0	100	KU981101.1
	*Streptomyces labedae* strain CTF14	1454	1454	100	0	100	EU294135.1
AIA-10	*Streptomyces variabilis* strain HBUM173496	1070	1070	99	0	92.01	EU841661.1

**Table 3:** Morphological characteristics of selected *Streptomyces* isolates on different media.

Isolates	Culture medium	Growth	Aerial mycelium	Substrate mycelium	Soluble pigment
SCA-11	SCA	Good	Grey-brown	Brown	None
	ISP-1	Good	White-yellow	White-beige	None
	ISP-2	Very good	White	Brown	None
	ISP-4	Good	White	Beige-brown	None

AIA-10	SCA	Good	Grey-Brown	Cream-light brown	None
	ISP-1	Good	White-yellow	White	None
	ISP-2	Very good	White	Cream-light brown	None
	ISP-4	Good	None	Brown	None

SCA-8	SCA	Very good	Brown	Yellow	Light-yellow
	ISP-1	Good	White	White-beige	None
	ISP-2	Very good	Grey	Cream-light brown	Light-yellow
	ISP-4	Good	None	Brown	None

SCA-5	SCA	Good	Grey-brown	Cream-light brown	None
	ISP-1	Good	White	White-beige	None
	ISP-2	Very good	White	Cream-light brown	None
	ISP-4	Good	White	Beige-brown	None

**Table 4:** Microscopy morphological, physiological, and biochemical characteristics of selected *Streptomyces* isolates.

Properties	SCA-5	SCA-8	SCA-11	AIA-10
*Microscopy morphological characteristics*				
(1) Sporophore morphology	Retina-culiaperti	Spira	Retina-culiaperti	Recti-flexibiles

*Physiological characteristics*				
(1) pH range for growth	5–9	6–9	5–9	5–9
(2) Optimum pH for growth	7	7	7	7
(3) NaCl tolerance	6%	6%	6%	4%
(4) Hemolytic activity	−	−	−	−

*Biochemical characteristics*				
(1) Citrate utilization	+	+	+	+
(2) H_2_S production	−	−	−	−
(3) Indole production	−	−	+	−
(4) Voges proskauer	+	+	+	+
(5) Starch hydrolysis	+	+	+	+
(6) Enzyme production				
Urease	+	+	−	−
Gelatinase	+	+	+	+
*β*-Galactosidase	−	−	−	−
Arginine dihydrolase	+	−	−	−
Lysine decarboxylase	+	−	−	−
Ornithine decarboxylase	+	−	−	−
Tryptophan deaminase	+	+	+	+
(7) Utilization of carbon source				
Glucose	−	+	+	−
Mannitol	+	−	−	−
Inositol	−	−	−	−
Sorbitol	+	−	−	−
Rhamnose	−	+	−	−
Saccharose	+	+	+	+
Melibiose	−	−	−	−
Amygdalin	+	+	−	+
Arabinose	+	+	+	+

+: positive reaction, −: negative reaction.

**Table 5:** Inhibition zone (mm) of the metabolite-crude-extract (10 mg/mL) by the disc-diffusion method.

Crude extract	Inhibition zone (mm)			
	*S. aureus*	*B. cereus*	*E. coli*	*P. aeruginosa*
SCA-5	9.7 ± 2.38^a^	9.5 ± 1.33^a^	9.0 ± 2.69^a^	8.4 ± 0.70^a^
SCA-8	11.1 ± 1.42^a^	9.8 ± 1.37^ab^	9.8 ± 4.70^a^	12.3 ± 1.80^b^
SCA-11^*∗*^	13.3 ± 1.33^a^	11.6 ± 1.36^b^	11.7 ± 1.46^a^	15.3 ± 1.65^c^
AIA-10	22.2 ± 3.08^b^	17.8 ± 0,46^c^	18.5 ± 0.87^b^	21.3 ± 1.63^d^
Control (+)	23.8 ± 0.47^c^	30.7 ± 0.07^d^	22.4 ± 0.20^c^	10.4 ± 0.17^e^
Control (−)	0 ± 0	0 ± 0	0 ± 0	0 ± 0

^*∗*^As reported by Kurnianto et al. (25); value was mean ± SD (standard Deviation) from three replication; Control (+): ampicillin 10 *µ*g/mL; Control (−): DMSO 2%; (a, b, c, d, and e): different letters in the same column represent significance.

**Table 6:** MIC and MBC value of the metabolite-crude-extract (10 mg/mL).

Crude extract	MIC (mg/mL)				MBC (mg/mL)			
	SA	BC	EC	PA	SA	BC	EC	PA
SCA-5	5	5	5	10	10	10	10	10
SCA-8	10	10	10	10	10	10	10	10
SCA-11	2.5	2.5	2.5	2.5	5	5	5	5
AIA-10	2.5	2.5	2.5	2.5	5	5	5	5
Control (+)	0.035	0.035	0.07	0.62	0.07	0.07	0.15	1.25
Control (−)	−	−	−	−	−	−	−	−

(−): no activity; Control (+): ampicillin; Control (−): DMSO 2%; SA: *S. aureus*; BC: *B. cereus*; EC: *E. coli*; PA: *P. aeruginosa*.

## Data Availability

All datasets generated or analyzed during this study are available upon reasonable request from the corresponding author
